# Supplementary material for: Are carbon emissions trading and green financial instruments synergistic? -Comprehensive quantitative research based on content analysis
Source: PLoS One. 2024 Mar 7;19(3):e0298601. doi: 10.1371/journal.pone.0298601 (PMC10919601; doi:10.1371/journal.pone.0298601)
Supplement: S1 Data — (ZIP) [file pone.0298601.s001.zip › Data/地级市Z_F工作报告.zip!/║╙─╧/░▓╤⌠╩╨/└┤╫╘╣1⁄2╓┌║┼í╛┬φ┐╦╩2╛▌═°í┐_2005.docx]

各位代表：

　　现在，我代表市人民政府，向大会作政府工作报告，请予审议，并请各位政协委员提出意见。

　　一、2005年及十五时期政府工作回顾

　　2005年，市人民政府在省委、省政府和市委的正确领导下，紧紧围绕“四路并进、五力同驱”及构建和谐安阳的要求，开拓进取，扎实工作，较好地完成了市十一届人大二次会议确定的各项目标任务。

　　——经济实力进一步增强。初步统计，全市生产总值556亿元，增长14.8%，其中，一产增长7.1%，二产增长18.2%，三产增长12.8%。人均生产总值突破10000元。

　　——工农业生产持续增长。限额以上工业实现增加值220亿元，增长22%。全市粮食总产量254万吨，增长3.3%，是历史上第二个高产年。

　　——质量效益明显提升。全市限额以上工业企业实现利润28.8亿元，增长60.4%，创历史最好水平。地方财政一般预算收入完成29亿元，增长25.9%。

　　——三大需求显著增长。全社会固定资产投资完成230.8亿元，增长50.2%。社会消费品零售总额145.6亿元，增长14.5%。进出口总额7.3亿美元，增长16%；实际利用外资2832万美元，增长51.2%。

　　——就业再就业超过预期目标。新增城镇就业人员10.3万人，下岗失业人员实现再就业5.3万人，其中安置“4050”人员1.4万人；城镇登记失业率控制在4%。

　　——居民收入不断增加。城镇居民人均可支配收入8823元，增长11.6%；农民人均纯收入3220元，增长14%。金融机构各项存款余额523.2亿元，比年初增加60.9亿元。

　　——县域经济快速发展。五县(市)生产总值完成359.8亿元，增长18.5%，高于全市平均水平；财政一般预算收入突破10亿元，增长26.9%。

　　2005年各项目标任务的胜利完成，标志着“十五”时期我市经济社会发展实现了阶段性突破。综合实力再上新台阶。国民经济连续五年实现了较快的增长速度和较高的增长质量，总量比“九五”末净增300亿元，年均增长12.8%，高出全省平均水平1.3个百分点，高出全国4.9个百分点。结构调整取得新进展。二、三产业比重上升到84%，比“九五”末提高4.8个百分点。高新技术产业增加值占全市生产总值的比重达17.5%。基础设施和基础产业建设实现新突破。全社会固定资产投资累计完成639.3亿元，是“九五”时期的2.4倍。新建改建干线公路580公里，完成和在建高速公路186.8公里。对外开放取得新成效。五年累计完成进出口总额22.6亿美元，年均增长28.8%，其中出口总额完成11.4亿美元，是“九五”时期的3.9倍，实际利用外资2.15亿美元，比“九五”时期净增2400万美元。人民生活水平有了新提高。城镇居民人均可支配收入和农民人均纯收入分别比“九五”末增加4428元和1091元，年均增长12.8%和9%；居民消费价格总水平保持基本稳定，年均上涨1.8%。社会事业又有新进步。科技对经济增长的贡献率43%，比“九五”末净增3个百分点；卫生机构病床数12564张，比“九五”末增长37.5%；人口自然增长率4.9‰，低于全国全省平均水平；全民环保意识不断提高，生态建设和环境保护成效明显。经过“十五”时期的发展，我市经济社会发展站在了新的战略起点上，进入了全面提升产业层次、加快经济转型、促进统筹协调发展的阶段。

　　回顾“十五”时期尤其是近年来的工作，我们的主要做法和体会是：

　　(一)实施“四路并进”，经济社会实现又快又好发展。我们紧紧抓住新一轮经济增长机遇，凝聚全市人民的智慧和力量，坚持不懈地推进了“农业立市、工业强市、三产富市、文化兴市”。农业基础地位进一步巩固。实施了四大兴农计划、三大惠民工程、十大农业产业链和十大增效技术。优质粮食和畜产品生产加工基地建设成效显著，优质强筋小麦面积达297万亩，占小麦播种面积的70%，畜牧业产值占农业总产值的比重达36%，比2000年提高了6个百分点。全面启动了“35411”农业产业化经营带动计划，全市农业产业化龙头企业达94家，培育农业产业化示范乡镇和特色农村经济先进乡镇29个，新建300万元以上的畜禽规模养殖场31个，新建、完善畜牧密集区70个；新增高效农业面积21.9万亩，完成退耕还林和四荒拍卖造林15万亩。开工建设了小南海泉域综合治理、马家岩水库、洹河河道治理等一批重点水利工程，退耕还林、太行山绿化等重点工程稳步推进，农村和农业基础设施不断完善。大力发展劳务经济，去年外出务工人员70余万人，劳务收入已成为农民增收的主要渠道。工业在调整中快速发展。狠抓了“工业振兴工程”，加强了36个工业项目和12个工业园区建设。安钢120吨转炉及炉卷轧机、汤阴华龙面粉等一批重点工业项目建成投产。骨干企业竞争力显著增强，年销售收入亿元以上的企业达102家，其中安钢突破200亿元，安彩等9家企业突破10亿元，成功申报了国家(安阳)显示器件产业园和省汽车零部件工业基地。运用高新技术和先进适用技术改造提升了钢铁、机械、纺织、医药等传统产业，加快了煤炭行业整合重组。认真落实国家宏观调控政策，加大了安林公路环境污染综合整治力度，压缩淘汰了一批落后生产能力，关闭小钢铁、小水泥等浪费资源的企业192家。第三产业逐步壮大。积极发展文化旅游、生态旅游和红色旅游，2005年接待国内外游客980万人次，旅游总收入达35.4亿元，创历史新高。金融、现代物流、信息、咨询等现代服务业快速发展，住房、通信等消费热点进一步升温，沃尔玛、大华物流等一批大型商贸集团和物流集团落户我市。文化事业取得新的成效。成功举办了4届殷商文化旅游节，先后在北京、上海等15个城市和地区进行红旗渠精神巡回展，深入开展了创建文化先进县(市)、民间艺术基地等活动。文化设施建设有了较大进展，建成了市殷墟博物馆，基本建成了市博物馆、图书馆等重点文化设施，拥有县级图书馆5个、博物馆3个、文化馆9个。广播电视和新闻出版进一步繁荣，创作了《红旗渠》、《商颂》、《甲骨惊魂》等一批文艺作品。

　　(二)不断优化投资结构，城乡面貌发生明显变化。我们从加强中心城市带动作用、提高县(市)经济发展水平入手，加大了城乡发展力度。着力抓好重点项目建设。“十五”期间，全社会固定资产投资增长34.9%，党政综合楼、汽车客运东站、东区污水处理厂等一批项目建成使用。积极鼓励、引导社会资金投入，2005年民间投资占全社会固定资产投资的比重达54%。投资结构进一步优化，工业投资和基础设施投资比重分别达76%和20%，均比2000年有大幅提高。加强了中心城市建设。顺利完成了城区行政区划调整，市区面积由247平方公里扩大到543.6平方公里，市区人口由75万增加到102万，城市框架不断拉大。加大了旧城及街景改造力度，新增、改造城市道路237公里，完成小巷治理387条；水、电、气、暖等基础设施功能增强，新增城市园林绿地1333万平方米，建成区城市绿化覆盖率达到34.5%。加快城镇化进程。按照“三头并举、突出重点、协调发展”的要求，完善城镇功能，增强产业聚集，推进了城镇化进程。2005年城镇化率达到32%。大力改善交通条件，完成投资61亿元，新建、改扩建公路3436公里。积极开展“一申四创”活动。殷墟申报世界文化遗产已顺利通过联合国专家评估，成为2006年中国唯一的文化遗产申报项目；成功创建了“中国优秀旅游城市”和“省级园林城市”，“省级文明城市”创建工作进展顺利，被省评为创建工作先进城市，创建“国家卫生城市”已通过省级初验。

　　(三)努力构建和谐安阳，经济社会协调发展。我们不断强化社会管理和公共服务职能，努力促进社会全面进步。大力发展各项社会事业。继续实施“科教兴安”、“人才强市”战略，开展了创建全国科技进步先进城市活动，连续4年举办了全国特种养殖种植品种展示交流会。五年累计获得省科技进步奖32项，市科技进步奖188项，授权专利726项。以安阳师院、安阳工学院专升本为契机，加快推进大学园区建设，普通高校在校生达2.9万人，年均增长28.3%；新建、改扩建高中阶段学校20余所，高中阶段入学率达到53%。高度重视人才工作，五年引进博士、硕士研究生262名。城乡医疗卫生条件不断改善，建立健全了卫生监督、疾病控制和医疗救治等公共卫生体系，开展了新型农村合作医疗试点。广泛开展全民健身活动，竞技体育水平不断提高。精神文明和民主法制建设得到加强。深入开展了保持共产党员先进性教育活动，选派五批7700多名干部进驻1848个行政村开展帮扶工作。继续推进基层民主政治建设，顺利完成了第四、第五届村委会换届选举，城市社区建设成效明显。自觉接受人大法律监督、工作监督和政协民主监督，五年累计办理人大代表建议1109件和政协提案2313件。廉政建设和反腐败斗争取得新成效。深入开展了双拥工作，连续三次荣获国家双拥模范城，连续六次荣获省双拥模范城，军政军民团结不断巩固。加强了信访工作和社会治安防控体系建设，信访案件结案率大大提高，各类违法犯罪活动得到有效遏制，处理突发事件能力不断增强，连续两年被评为全省社会治安综合治理先进市。深入开展安全生产专项整治活动，安全生产状况明显改善。全面推进了社会化消防安全防范体系建设，连续13年被评为省先进消防支队，连续18年未发生特大火灾事故。去年我市被评为全国人民防空先进城市，并由国家三类人防重点城市提升为国家二类人防重点城市。依法保护妇女权益，老龄和关心下一代工作健康发展。民政、统计、审计、地震、史志、档案、无线电管理、经济研究、民族宗教等工作也都取得了新成绩。

　　(四)着力推进改革开放，发展活力明显提升。我们强力把改革向纵深推进、向广度拓展，努力扩大对外开放，经济增长活力进一步增强。积极推进各项改革。以产权制度改革为核心，推动了国有企业改革，国有企业改制面达90%以上。完善了支持非公有制经济发展的政策措施，不断优化发展环境，非公有制经济增加值占全市生产总值的比重达46%。取消了农业特产税，提前一年免征农业税，全市农民减负增收2.3亿元。完成了粮食流通体制改革，国有及国有控股粮食购销企业由132家减少到39家，粮食购销市场主体多元化格局初步形成。加快财税体制改革步伐，初步形成了公共财政框架。农村信用社改革进展顺利。积极稳妥地推进了科技、教育、文化、卫生、城市公用事业等领域改革。进一步扩大对外开放。充分借助红旗渠精神巡回展等平台，大力开展招商引资工作，近两年全市引进域外项目826个，到位资金79.3亿元。外事侨务工作不断加强，先后与日本草加市、加拿大莱桥市等5个国外城市建立了友好城市关系，开展了一系列国际交流与合作活动。在中央电视台成功制作播出了《让世界了解你》安阳专题节目，我市的国际影响力和知名度不断提升。

　　(五)坚持以人为本，群众享受了较多的发展成果。我们投入更多的精力和财力，着力解决群众最直接、最关心、最现实的问题，力求让人民群众得到更多实惠。认真抓好政府每年向全市人民承诺的十个方面重要实事，较好地解决了50个方面、120余件群众普遍关注的难点、热点问题。积极落实就业再就业扶持政策，城镇新增就业人员20.6万人次，下岗失业人员再就业7.7万人，其中“4050”人员2.6万人。强化了养老、失业、医疗、工伤四项保险的扩面征缴，加强了“两个确保”和城市“低保”工作，全市406.9万人次纳入低保。筹措资金2.4亿元用于农村中小学危房改造，累计改造农村中小学1012所，消除D级危房52.9万平方米，提前两年超额完成改造任务。投入资金6600万元，建成覆盖全市农村中小学的现代远程教育网络。认真落实农村义务教育“两免一补”政策，资助农村贫困家庭中小学生4588万元。投入资金1500万元改造了30所乡镇中心卫生院。利用侨资兴建希望小学9所。加大了扶贫救灾力度，五年共有18.4万农村贫困人口脱贫，解决了西部缺水区25.9万人和东部苦水区25.5万人的饮水困难。新增农村沼气用户5万余户，有效地改变了农村庭院面貌。完成了农村电网改造，实现城乡居民生活用电同网同价。推进了建筑领域拖欠工程款和农民工工资清理工作，较好地维护了务工人员的合法权益。

　　(六)积极转变职能，政府自身建设得到加强。我们坚持开拓创新，着力提高行政能力和服务水平。顺利完成了政府机构改革，市政府机构减少26%，行政编制总体精简24.6%；乡镇机构改革任务如期完成，分流4427人，清退1436人，乡级领导职数减少了325个。贯彻实施了《行政许可法》，认真清理行政许可项目和主体，行政审批项目由1603项减少到319项，减幅达80%，推行并联审批、代办代理制度，建立了投资建设项目审批“绿色通道”。简化了行政审批程序，在全省率先开展了建设服务型政府工作，建成行政便民服务中心、劳动保障服务中心、行政效能监察中心、人才开发服务中心和财政国库支付中心，推出了政府电子政务内网、多媒体信息亭等一系列服务新举措。探索完善各种应对突发事件的预案和应急机制，提高了政府预防和处置突发公共事件的能力；推行了相对集中行政处罚权工作；进一步优化经济发展环境，严肃查处了影响企业环境的刑事案件2100多起；开通了市长便民公开电话、效能监督热线，开播了《阳光对话》电视栏目，完善了政府新闻发布制度，畅通了领导与群众、政府与企业沟通的渠道。

　　各位代表，过去的五年，是我市物质文明、政治文明和精神文明全面进步的五年，也是我市综合竞争力提升较快、城乡面貌变化较大、人民群众收益较多、现代化水平不断提高的五年。这些成绩的取得，是市委正确领导的结果，是市人大、市政协大力支持的结果，是全市人民同心同德、团结奋斗的结果。在此，我代表市人民政府，向奋斗在各条战线的广大工人、农民、知识分子、干部、解放军指战员、武警官兵、政法干警，致以崇高的敬意！向给予政府工作大力支持的人大代表、政协委员、各民主党派、工商联、人民团体以及社会各界人士，表示衷心的感谢！向所有关心、支持我市现代化建设的离退休老同志和港澳台同胞、海外侨胞、国际友人，表示诚挚的谢意！

　　在肯定成绩的同时，我们也清醒地看到，经济社会发展和政府工作还存在着一些矛盾和问题，主要是经济增长方式粗放，产业结构不尽合理；对外开放程度较低，域外资金进入规模不大；经济发展与资源环境的矛盾日趋尖锐，改革的难度不断增大；一些地方、部门和政府工作人员办事效率低，为群众、为基层、为企业服务的主动性不够，形式主义、奢侈浪费现象依然存在，思想不解放，市场意识淡薄，抓改革促发展的紧迫感和责任感不强等。对此，我们一定高度重视，在今后的工作中认真加以解决。

　　二、“十一五”时期的总体要求和战略重点

　　“十一五”时期是我市必须紧紧抓住并且可以大有作为的战略机遇期，也是我市实现全面建设小康社会、加快建设豫北区域性中心强市的关键阶段。世界经济的增长态势、国际产业的快速转移，有利于我们改革开放、招商引资。国家实施中部崛起战略，在产业发展、重大基础设施建设等方面加大支持力度，有利于我们加快经济结构调整。我市的经济社会发展进入新阶段，基础设施日益完善，体制机制不断创新，发展环境进一步优化，全市上下想发展、谋发展的氛围愈加浓厚，这些都有利于我市在新的战略起点上加快发展。我们一定要按照“决战十一五、提高总实力、转型上轨道、实现大跨越”的要求，进一步增强责任感、使命感，抢抓机遇，精心谋划，推动“十一五”经济社会发展再上新台阶。

　　“十一五”时期，我市经济社会发展的总体要求是：以邓小平理论和“三个代表”重要思想为指导，以科学发展观统领经济社会发展全局，认真贯彻党的十六大、十六届五中全会精神，坚持“四路并进”，加强自主创新，大力实施科教兴安、人才强市、开放带动和可持续发展战略，强力推动各项改革，大力发展非公有制经济、外向型经济和县域经济，全面提升综合实力和竞争力，着力构建和谐安阳，努力实现经济社会跨越式发展。我市经济社会发展的主要奋斗目标是：经济实力显著增强。全市生产总值年均增长12%，人均生产总值提前3年实现比2000年翻一番；财政一般预算收入高于生产总值增长；城镇化水平达到40%。开放型经济明显提升。五年累计利用外资2.5亿美元，2010年外贸进出口总额11.9亿美元；非公有制经济增加值比重达到55%。生态环境得到改善。单位生产总值能耗比“十五”末下降20%左右，森林覆盖率达到28%。社会事业全面进步。科技对经济增长的贡献率达到50%，教育水平进一步提高，文化建设取得突破性进展，新型农村合作医疗参保率达到90%。城乡居民生活水平明显提高。城镇居民人均可支配收入年均增长8%，农民人均纯收入年均增长7.8%；社会就业比较充分，社会保障体系比较健全，民主法制和精神文明建设进一步加强，和谐社会建设取得显著成效。

　　实现以上奋斗目标，要着力抓好以下战略重点：

　　(一)继续坚持“四路并进”，提高总体竞争力。“四路并进”是我市实现宏伟奋斗目标的基本途径和重要支撑，必须统筹兼顾，突出重点，加快推进。加快推进“农业立市”。把解决好“三农”问题作为全市工作的重中之重，统筹城乡经济社会发展，建立以工促农、以城带乡的长效机制，加快建设社会主义新农村。实施“百万亩优质高效农业开发”计划和“抓百区、带万户，创建畜牧大市”计划，继续实施“35411”产业化经营带动计划，重点打造粮食、棉花、花生、林果、畜牧、瓜菜菌等十大农业产业链，培育国家级农业龙头企业3家以上。继续抓好150万人的沼气建设和100万人的安全饮水工程。加强农村劳动力技能培训，引导富余劳动力向非农产业和城镇有序转移，“十一五”末，全市劳务输出达到100万人次。加快推进“工业强市”。把加快工业化进程摆在经济社会发展更加突出的位置，走新型工业化道路，大力推进工业振兴工程，整合和振兴机械、纺织、医药、建材工业，提升工业发展的档次。着力建好钢铁、食品、煤化工、电子、先进制造业五大工业基地。不断加大财政投入，增强自主创新能力，加强创新体系建设，加快用先进技术和高新技术改造传统产业步伐，促进科技与经济紧密结合。加快发展循环经济，大力推进资源节约，合理开发优势能源和矿产资源，提高资源利用效率，促使经济增长由主要依靠资源投入转向主要依靠自主创新和经济增长方式转变上来。加快推进“三产富市”。大力发展旅游业，进一步整合旅游资源，积极实施“1715”工程，打造豫北区域性文化旅游中心。大力发展旅游相关产业，积极开发具有地方特色的文化旅游商品，到“十一五”末，旅游总收入占全市生产总值的比重达到7%以上。加快发展现代物流业，重点搞好物流结点发展规划，把安阳建成与郑州中心城市相呼应，带动豫北地区，辐射河北、山西，连接山东、京津地区的重要物流枢纽。加快发展房地产业，积极发展社区服务、信息服务、中介服务和金融保险等服务业。加快推进“文化兴市”。把发展文化产业作为重要突破口，使文化产业成为我市国民经济新的增长点。繁荣文化艺术事业，推动文学艺术、新闻出版、广播影视等快速发展，不断涌现文化精品。加强文化设施建设，建成一批现代化文化建筑和群众文化基础设施。完成河南中国文字博物馆、安阳会展中心等文化设施建设。

　　(二)加快城镇化进程，着力建设宜居城市。按照建设现代化大城市的要求，优化城市发展布局，完善六大功能区建设，处理好文化名城、工业重镇和现代化城市的关系，在老城区和殷墟保护区体现历史文化名城特色，在东南新区体现现代化城市风貌，在工业园区体现工业大市的实力，确保城市整体形象实现“三年提升、五年大变”的目标。围绕争创国家卫生城市、国家园林城市的目标，加大环境治理和美化、绿化力度，疏浚水系，完成环城河综合治理和洪水河城区段综合治理工程，加快游园绿地建设，使安阳成为环境优美、生态良好、交通便利、生活舒适、人文和谐的宜居城市。加快构建现代化交通体系，建设长泰铁路安阳段和北京至郑州铁路客运专线安阳段，建成安林、安南、林长、大广和济东高速公路安阳段，形成南北以京珠高速，东西以安南、安林、林长高速构筑的“十字”型交通骨架。积极推进连接卫星城市的高等级外环线建设，拉大中心城市框架，完善市区边缘和市内立体交通网络，优化公交线路和场站布局，提高通行能力。

　　(三)强力推进改革开放，实现经济有序转型。以根本消除经济增长的体制性障碍为目标，力争在重点领域、关键环节取得实质性突破。继续加大国有企业和公用事业改革力度，完善国有资本有进有退、合理流动的机制，加快产权制度改革和国有产权转让。健全国有资产监督管理体系，确保国有资产保值增值。积极鼓励、支持、引导非公有制经济加快发展，进一步放宽市场准入和股权比例限制，逐步提高非公有制经济比重，使之成为推动我市经济社会发展的主要力量。坚持以开放促发展，形成更具活力的开放型经济体制，大力引进战略投资者，实现由主要依靠自身潜力向更加注重借助外部资金、技术、人才等资源的转变，加强同其他地市的横向联合和协作，实现优势互补、共同发展。

　　(四)全面建设和谐安阳，保持经济社会协调发展。紧紧围绕构建和谐安阳的总目标，更加关注民生，努力打造活力、有序、公允、诚信、平安和秀美安阳，促进经济与社会、人与自然协调发展。千方百计扩大就业，加快社区服务平台、就业服务网络建设，形成经济增长与就业扩大共进的发展模式，确保全市每年新增城镇就业人员5万人以上。建立和完善多支柱、多层次、广覆盖的社会保障体系，规范完善基本养老保险市级统筹。合理调节收入分配，不断提高城镇低收入者和广大农民的收入水平，扩大中等收入人群的比重，认真解决低收入人群的住房、医疗和子女就学等困难，努力缓解部分社会成员收入分配差距扩大的趋势。切实保护生态环境，坚持节约发展、清洁发展、安全发展和可持续发展，促进经济发展与人口、资源、环境相协调，“十一五”末万元工业增加值用水量降低16.7%，主要污染物排放量削减5%。加快发展科技、教育、文化、卫生、体育等社会事业，健全公共服务体系，促进人的全面发展和社会全面进步。创新社会管理体制，妥善协调各种利益关系，注重社会公平。加强民主法制建设，正确处理改革发展稳定的关系，确保社会大局稳定。

　　《安阳市国民经济和社会发展第十一个五年规划纲要(草案)》及其说明已印发各位代表，请予审议。

　　三、2006年主要目标和工作任务

　　2006年是“十一五”规划的开局之年，也是为全面建设小康社会奠定坚实基础的关键之年。今年我市经济社会发展的主要预期目标是：全市生产总值增长13%，工业增加值增长16%，财政一般预算收入增长13%以上，全社会固定资产投资增长20%以上，社会消费品零售总额增长13%，外贸出口增长12%，实际利用外商直接投资增长20%以上，居民消费价格总水平上涨3%，城镇居民人均可支配收入增长8%，农民人均纯收入增长6%，单位生产总值能耗降低5%左右，人口自然增长率控制在6‰以下，城镇登记失业率控制在4.5%以内，城镇新增就业人数6.5万人。

　　在实现上述目标的同时，今年将为全市人民办好十个方面的实事。(1)抓好粮食直补、良种补贴、购置大型农机具补贴工作；落实村级经费和村级干部报酬；完成国家大型优质小麦生产基地项目建设。(2)对农村义务教育阶段贫困家庭学生继续实施“两免一补”；提高农村中小学公用经费标准，建立农村中小学危房改造长效机制，抓好农村中小学D级危房改造；建设农村党员远程教育工程，建成覆盖全市的农村信息网；建成市区三所高中；筹建安阳职业技术学院。(3)完成20所乡镇卫生院建设任务；新型农村合作医疗试点县由1个增加到4个，试点县的参保农村人口每人每年财政补贴标准由20元提高到40元；确保村村有规定数量的卫生预防保健人员和经费；基本完成市传染病医院新建任务。(4)改造和建设农村公路338公里；开工建设汽车客运南站，新增和优化公交线路5条；实施市区东、南出口改造工程；开工建设市北外环经水冶、铜冶、陵阳工业区至姚村公路。(5)增加农村沼气用户4万户，在沼气推广成规模的县区建设沼气服务站；对卫河流域安阳段水污染进行治理；解决重污染高氟高盐地区8万人安全饮水问题。(6)提高城市居民最低生活保障标准，农村特困人口每人每月生活最低补助标准提高到15元；加强农村敬老院建设，提高五保户集中供养率；加大扶贫开发投入，完成3万农村贫困人口的脱贫任务。(7)做好农村劳动力转移培训和劳务输出工作，新增劳务输出10万人；切实解决进城务工农民户籍、子女入学等难点问题。(8)新增就业人员6.5万人，安置下岗失业人员3万人，其中安置“4050”人员1万人。(9)完善配套设施，完成殷墟申报世界文化遗产任务。(10)完成小巷治理工程；继续推进环城河治理；开发治理洪水河；逐步实行公厕免费开放；新建经济适用住房10万平方米。

　　围绕上述目标和实事，今年将重点抓好以下八个方面的工作：

　　(一)着力提高产业竞争力。以增强自主创新能力为核心，促进工业和服务业总量扩大、结构升级、经济转型、质量提高。着力发展优势产业。加大工业结构调整力度，以安钢建设千万吨优质钢基地为契机，引导钢铁企业实施联合。充分发挥安彩集团的龙头作用，推进莱必泰、凯地、凯瑞等企业发展，抓好国家(安阳)显示器件产业园建设，进一步提升在行业中的竞争优势。支持发展煤炭和煤化工项目，拉长煤化工产业链条。以创新发展为动力，着力打造知名品牌，提高自主创新能力，努力实现观念、管理、技术、产品和品牌创新，加快华康功能大豆蛋白纤维、宏源型钢无内胎工程机械车轮总成等重点创新项目的研究与开发。进一步抓好工业振兴工程。支持安钢、安彩、安烟、安电、安化、锻压、鑫盛机床等重点企业做大做强，力争新增销售收入超亿元企业30户，超5亿元企业10户，超10亿元企业3户。重点支持安钢年产350万吨热轧薄板、安彩液晶显示玻璃等重点项目建设，确保安烟“十五”技改工程、旺旺休闲食品、安化集团年产40万吨甲醇及6万吨甲胺、湖波水泥日产4500吨干法水泥、林丰铝业年产5万吨铝板带箔一期生产线等项目建成投产。大力发展循环经济。积极推进安钢、安化和开发区3个省循环经济试点工作。整合、提升现有各类工业园区，实现物流、能源流、技术集成和信息与基础设施共享。加快发展非公有制经济。以优势产业为基础，以大型骨干企业为龙头，抓好产业集群，打造名牌企业和产品。建立健全中小企业、非公有制经济服务体系，大力构建融资担保、人才培训、政策法律、管理咨询、技术创新、创业辅导、信息网络等服务体系。大力发展服务业。进一步整合旅游资源，培育黄金旅游线路，加强旅游配套设施建设，加快旅游产品开发。积极发展历史文化游、自然风光游、民族精神游。深入挖掘旅游资源，打造精品景区，把殷墟、羑里城、红旗渠、太行大峡谷、二帝陵等一批旅游景点做大、做优。加强物流基础设施建设，组建大型物流企业，抓好大华商贸城等重点物流项目。积极促进金融、咨询、法律、会展、房地产、信息产业等服务业健康发展。

　　(二)扎实推进社会主义新农村建设。围绕“生产发展、生活富裕、乡风文明、村容整洁、管理民主”的要求，完善和强化各项支农惠农政策，力争新农村建设取得良好开端。大力发展农村经济。继续实施四大兴农计划、三大惠民工程、十大农业产业链、十大增效技术。建设完善国家大型优质小麦生产基地，做大做强粮食产业。加大对龙头企业支持力度，培育重点龙头企业10家、农业产业化示范乡镇和特色农村经济先进乡镇30个左右、各类农村经合组织50个。实施高效农业开发和现代农业发展计划，新增瓜果菌菜等高效农作物种植面积15万亩以上，新建、扩建投资100万元以上的大中型畜禽养殖场20个、标准化畜禽养殖密集区30个。大力推广农作物良种、畜禽改良、果实套袋、秸秆还田、配方施肥等十大增效技术，抓好粮、棉、油、瓜菌菜、畜禽、林果等十大农业产业链条式开发。进一步加强农村基础设施建设。加强村庄建设规划，优化村庄布局，重点发展中心村，引导农民向中心村集中；加强村庄环境整治，大力开展村庄绿化，加快农村公路、农田水利、农村沼气、安全饮水等乡村基础设施建设，改善农村生活环境和村容村貌。建设一批大中型农产品加工基地示范区、农产品专业批发交易市场、农林生态示范区，配套建设300万亩标准化生产基地，不断完善粮食、供销以及农业人才技术信息服务网络。全面推进农村综合改革。加快农村中小型水利设施管理体制改革、“四荒”拍卖造林改革。深化农村义务教育和县乡财政体制改革，化解乡村债务，巩固农村税费改革成果。积极推进农村土地流转、金融体制、流通体制等改革。

　　(三)不断加快城镇化步伐。逐步形成以中心城市为核心，小城镇为骨干，县(市)城和重点镇为依托的城镇化体系。加强中心城区建设。完成城市总体规划修编报批和各类专业专项规划的编制工作。加强规划管理动态监测，维护城市规划的权威性和严肃性。加快安东新区基础设施建设步伐，成立安东新区建设办公室，对安东新区规划、开发和建设实行统一管理。支持发展高层住宅和公共建筑，限制多层住宅，充分利用空间资源。加大老城、旧城及街景开发改造力度，加快城中村改造步伐。完善城市供水、供气、供热、污水和垃圾处理系统，加快市区路网建设，重点建设中华路、平原路等道路。加快洹河风光旅游带开发，建成愉字园，启动京珠生态园建设。积极推进城乡一体化试点。四个城区率先做好试点工作，在城市基础设施、社会保障体系、环卫体制、医疗卫生保障、文化教育、村改居、城乡交通等方面转换体制，强化对接，统筹城乡和谐发展。改革城镇户籍管理制度，逐步废除农业和非农业户口二元制管理模式，进一步加快城中村及近郊无地少地居民转户步伐。大力发展小城镇。规划整合水冶、柏庄、白壁、宝莲寺等卫星城的经济、社会和文化资源，逐步实现交通、通讯、金融同城化。建立小城镇建设多元投融资机制，改善小城镇投资环境。完善公共服务和基础设施，增强产业支撑，提高重点镇的聚集度和辐射带动能力。健全客运场站和乡镇等级客运站体系，完成50个乡镇客运站建设。发展壮大县域经济。落实和完善各项扶持政策，市财政安排2000万元专项资金，支持县域经济发展。促进永兴特优钢改造、健丰饼干、林州汽车零部件生产线、星河植物油、华康大豆纤维内衣生产线等项目建设。引导和支持县域发展特色工业园区，抓好柏庄纺织、水冶冶炼、姚村汽配、陵阳科技、白道口电缆、汤阴亚新钢铁等工业小区和产业集群建设。

　　(四)强力推进重大项目建设。优化投资结构，提高投资效益，加快推动重大项目建设，夯实发展基础。加强综合交通体系建设。完善高速公路网，增强互通能力。建成大广高速安阳段、安林高速，加快安南高速、济东高速安阳段建设进度，开工建设林长高速和安新高速拓宽改造工程。改建省道219永定线安阳段、307杨小线安阳段等4条干线公路和3座大中桥梁。积极推进能源基础建设。以大型煤炭企业为骨干，建设一批现代化大中型矿井。加快电网建设，建成洹安500千伏变电站和内黄220千伏输变电工程。积极做好大唐二期扩建、广源热电联产、宏源秸秆发电、煤矸石综合利用等项目的前期工作，争取早日开工建设。加快开发和利用煤层气，大力发展焦化煤气和高炉煤气。继续加强城镇基础设施建设。新增城市绿地面积100万平方米，推进城镇垃圾处理厂建设。建成西区污水处理厂，加快林州、滑县、汤阴、内黄污水处理厂和市区宗村污水处理厂及管网配套工程建设，全市医疗废物集中处理中心建成投运。进一步推进大型水利基础设施建设。继续实施“829”重点水利工程，全面开工建设南水北调中线安阳段，加快马家岩水库建设，完成红旗渠灌区节水续建配套和洹河河道治理工程年度目标，积极开展滑县、内黄生态恢复补源工程前期工作。

　　(五)进一步加大改革开放力度。全面推进各项改革，力争在重要领域和关键环节实现新的突破。强力推动国企改革。加快国有企业公司制改革、引进战略投资者和分离企业办社会步伐。市属国有工业企业基本实现产权多元化，国有大中型企业基本实现主辅分离，完成分离企业办学校任务。规范企业改制程序，切实保护职工合法权益。加快财政金融体制改革。合理划分市县乡政府的财权和事权，调整、规范三级政府间的收支关系。落实和完善部门预算、国库集中支付、政府采购和收支两条线管理制度。支持农村信用社改革，确保10.3亿元专项央行票据全额兑付。推进城市信用社改制，组建市商业银行。举办多种形式的银企洽谈活动，进一步拓宽银企合作领域。全面推进其他领域的改革。加快行政管理体制改革，减少和规范行政审批。深化环卫和园林体制改革，建立和完善“两级政府、三级管理、四级网络、全民参与”的城市管理机制。积极推进文化体制改革，加快国有文化企业公司制改造和经营性文化事业单位转企改制步伐。进一步推进卫生、科技、教育、交通、城建、城市公用事业等改革。继续实施开放带动战略。进一步扩大利用外资规模，抓好专项和专业性招商工作。发挥开发区和各类工业园区作用，依托骨干企业、优质资产和重大项目，重点吸引跨国公司、国内外高科技企业和现代服务企业投资。认真组织参加各类招商引资活动，切实加强签约项目跟踪落实，提高招商实效。优化进出口商品结构，尽快培育一批新的出口强势企业和拳头产品。抓好境外大型工程承包和对外劳务合作。加强与长三角、珠三角、环渤海经济圈以及中部省份地市的经济技术交流与合作。充分发挥友好交往和经济联络主渠道作用，积极筹办世界韩氏宗亲会。

　　(六)加快发展各项社会事业。进一步加大社会事业投入，努力促进经济与社会协调发展。大力发展文化事业。加强文化基础设施建设，开工建设河南中国文字博物馆，办好殷商文化旅游节等节庆活动。建立健全公共文化服务体系，实行文化信息资源共享。推进社区文化、村镇文化、企业文化、校园文化、军营文化建设。积极发展科技、教育、卫生、体育等社会事业。继续实施科教兴安战略，加大科技投入，推进科技攻关和共性关键技术开发。发展农村教育，切实保障义务教育经费，解决好城市低收入群众子女上学问题。加快发展职业教育、高等教育。继续抓好国家卫生城市创建工作。大力发展农村医疗卫生事业，推进社区卫生服务，加强医院管理，逐步解决群众看病难、看病贵的问题，开工建设市中医药学校新校区。广泛开展全民健身活动，完善业余训练体制，完成射击馆建设。稳定低生育水平，加大性别比治理力度，提高出生人口质量。加强环境保护和生态建设。加大环保基础设施建设力度，严格执行建设项目环保优先审批、环境影响评价等制度。抓好洹河、汤河、卫河、洪水河、金堤河污染治理。巩固安林公路综合整治成果，严防污染企业反弹。落实生态保护规划，切实加强生态建设，组织好退耕还林、太行山绿化、防沙治沙等重点生态工程。认真开展第二次全国农业普查。继续做好民政、审计、人防、地震、档案、史志、无线电管理、外事侨务、经济研究等工作。

　　(七)积极推进和谐安阳建设。以人为本，关注民生，让人民群众得到更多实惠。认真抓好就业再就业工作。多渠道开发就业岗位，支持发展社区就业和灵活就业。继续做好高校毕业生、进城务工农民、被征地农民以及退役军人的就业和安置工作。进一步完善和落实小额贷款、税费减免等再就业优惠政策，小额担保贷款完成6000万元以上，国有企业下岗职工再就业扶持政策再延长三年，并根据实际逐步扩大到城镇集体企业下岗职工。巩固和完善社会保障体系。加快建立就业与失业保险、城市居民最低生活保障的联动机制。完善城镇职工基本养老保险制度，确保企业离退休人员基本养老金按时足额发放。加强城镇基本医疗保险和失业、工伤、生育保险制度建设。关注弱势群体，建立弱势群体帮扶救助机制，充分尊重和保护弱势群体的合法权利，进一步完善城市居民最低生活保障制度，实现动态管理下的应保尽保。继续推进农村最低生活保障制度试点工作，完善农村特困户救助和五保户生活保障制度。高度重视安全生产。巩固小煤矿整顿关闭成果，加强煤矿、非煤矿山、危险化学品、道路交通、烟花爆竹及消防安全等领域和人员密集场所的专项整治，全面落实安全生产责任制和责任追究制，坚决遏制重特大事故发生。认真做好社会稳定工作。加强矛盾纠纷排查调处，认真做好人民群众来信来访，完善社会稳定预警体系和应急处置机制，积极预防和妥善处置群体性事件。进一步加强社会治安综合治理，强化平安建设，严厉打击严重暴力犯罪、团伙犯罪和多发性侵财犯罪，提高刑事案件破案率。加强技防工程、城市报警与监控系统建设，提高安全防范能力。加强精神文明建设。切实加强理想信念教育和思想道德建设，大力宣传、弘扬红旗渠精神。深入推进公民道德建设，倡导公民基本道德规范。继续开展科教、文体、法律、卫生、道德规范“五进社区”活动。广泛开展家庭和美、邻里和睦、人际和谐的“三和行动”。加强未成年人思想道德建设，抓好生理、心理、伦理“三理教育”。深入开展国防教育，健全国防动员体系。做好双拥共建工作，严格落实优抚安置政策，关心支持驻安部队和民兵预备役建设，巩固和加强军政军民团结。全面推进民主法制建设。坚持依法治市，提高全民法制素质。严格执行人大及其常委会的决议、决定，自觉接受其工作监督和法律监督，支持人大代表依法行使职权。接受人民政协的民主监督。认真办理人大代表建议和政协委员提案。广泛听取各民主党派的意见，发挥工会、共青团、妇联等人民团体的桥梁纽带作用。积极做好法律援助工作。依法保护妇女儿童、残疾人、老年人和未成年人的合法权益。全面贯彻党的民族宗教政策，做好民族宗教工作。

　　(八)切实加强政府自身建设。强化服务意识，不断提高行政能力和服务水平。继续推进依法行政。认真贯彻公务员法、行政许可法和全面推进依法行政实施纲要，更新行政理念，完善行政程序，规范行政行为。加快建立责权明确、保障有力的行政执法体制。强化行政复议和对行政执法的监督，实行行政执法责任制和执法过错追究制。完善行政管理决策机制，健全与群众利益密切相关的重大事项决策公示、听证和专家论证制度，推进行政管理决策的科学化、民主化。进一步加强服务型政府建设。积极整合行政资源，有效降低行政成本，切实提高行政效率。大力推进政务公开，从人民群众普遍关心、涉及群众切身利益的问题入手，在公共服务部门和单位全面推行办事公开制度。加强政府社会管理和公共服务职能，健全完善行政便民服务中心等政府综合施政平台。继续大力精简会议、文件，坚决改进会风、文风，严格控制和规范各种检查、评比、达标活动。带头厉行节约，反对奢侈浪费，建设节约型机关。全面推行经济责任审计和政府投资绩效审计。严格落实党风廉政建设责任制，坚持廉洁从政。不断优化经济发展环境。进一步整顿和规范市场经济秩序，建立健全整治企业生产经营环境长效机制。切实纠正损害群众利益的不正之风，坚决查处“四乱”和“吃拿卡要”行为。加大对破坏经济秩序违法犯罪行为的惩治，严厉打击制售假冒伪劣商品等违法犯罪活动。继续做好减负治乱工作，规范各种收费行为，保护市场主体的合法权益。

　　各位代表！

    我们正站在一个新的战略起点上。回顾过去，我们感到欣慰和自豪；展望未来，我们充满信心和希望。让我们高举邓小平理论和“三个代表”重要思想伟大旗帜，紧密团结在以胡锦涛同志为总书记的党中央周围，在市委的正确领导下，同心同德，锐意进取，为开创我市经济社会发展新局面、建设豫北区域性中心强市而努力奋斗！
